# Sea surface surveys for microplastic and floating marine macro litter items in coastal waters of Cabrera Archipelago Maritime Terrestrial National Park

**DOI:** 10.1007/s11356-023-27710-x

**Published:** 2023-06-02

**Authors:** Montserrat Compa, Carme Alomar, Beatriz Rios-Fuster, Valentina Fagiano, Salud Deudero

**Affiliations:** Centro Nacional Instituto Español de Oceanografía, Centro Oceanográfico de Baleares, Consejo Superior de Investigaciones Científicas (IEO-CSIC), Muelle de Poniente s/n, 07015 Mallorca, Spain

**Keywords:** Floating plastic litter, Microplastics, Spatial distribution, Marine protected area, Western Mediterranean Sea

## Abstract

This study is aimed at identifying and comparing microplastics and floating marine litter along the sea surface in the marine protected area of Cabrera Archipelago Maritime Terrestrial National Park (Cabrera MPA) in the Balearic Islands. A total of 52 net surveys and 22 visual surveys were carried out between July and August in 2019 and 2020. The abundance of microplastic (MP) items was highest in the southern and eastern regions, with an average of 381,244.4 ± 1,031,082.8 items/km^2^ weighing an average of 927.1 ± 2731.4 g/km^2^. Most of these items were < 5 mm (81%) in size and were mainly composed of polyethylene and polypropylene (98%). In terms of floating marine macro litter (ML) from visual surveys, an average of 2028 ± 2084 items/km^2^ were observed. In this case, the majority of the ML items were plastic pieces (69%) measuring 2.5 to 50 cm. Furthermore, ML quantified by visual surveys was an order of magnitude higher than in similar studies carried out on large vessels, highlighting the importance of vessel height and speed for identifying the smallest size fractions (81%). The results of this study document the intensity of MPs and ML, primarily plastic, in coastal waters, and provide a baseline for management efforts to mitigate floating litter, in addition to raising awareness of the transferability of marine litter from other regions.

## Introduction

Currently, an estimated 170 trillion pieces of plastic are floating on the surfaces of global oceans (Eriksen et al. 2023). According to the United Nations Environment Programme (UNEP), the majority of marine litter, of which > 80% is estimated to be plastics, originates from land sources including agriculture, cities (e.g., building, construction, and transportation), and sewage and wastewater among others, while the main sources from the sea include fisheries and aquaculture, shipping, offshore operations, and ship-based tourism (Maes et al. 2021). From the numerous sources, plastics can enter the marine environment impacting habitats through several pathways such as sewage, storm water, runoff, and rivers (Veiga et al. 2016). The alarming amount of plastic litter in marine ecosystems has led to several European directives, such as the Waste Framework Directive (European Commission 2018) and the Marine Framework Strategy Directive (European Commission 2008) to prevent and reduce waste generation and the adverse impacts it can cause.

Plastic marine litter is usually classified as microplastics (< 5 mm), mesoplastic (5 to 25 mm), and macro litter (> 25 mm). In terms of abundance in the marine environment, microplastic items are estimated to consist of 92% of items floating on the sea surface globally (Eriksen et al. 2014). In the Mediterranean Sea, model simulations estimate that there is an average of ∼ 3760 tones of plastic floating on the sea surface (Tsiaras et al. 2021) which is slightly higher than empirical observations that range from 756 to 2969 tones (Cózar et al. 2015). Within the Balearic Islands in the western Mediterranean basin, some of the highest concentrations of small plastic particles floating along the sea surface have been found with an average of 900,324 ± 1,171,738 items/km^2^ weighing an average of 1165.72 ± 2335.84 g/km^2^ (Ruiz-Orejón et al. 2018), highlighting their prevalence in marine ecosystems.

Plastic is transported throughout marine environments across biotic (i.e., biofouling) and abiotic factors (i.e., winds, currents) as it is transported from the sea surface to beaches or from coastlines to the water column, reaching the sediments, and putting at risk the biota that inhabits all compartments of the marine environment. The presence of plastic in the marine environment can cause several biological and economic risks (Compa et al. 2019; Newman et al. 2015). In terms of biological risks, plastic litter can cause physical damage that can lead to mortality when ingested (Wilcox et al. 2018), induce oxidative stress in mussels and fish (Capó et al. 2021a, b; Solomando et al. 2020), and transfer hazardous chemicals such as organochlorine pesticides and polychlorinated biphenyls (Rios-Fuster et al. 2021a), all of which could potentially alter fish behaviour (Rios-Fuster et al. 2021b). Furthermore, plastic debris can act as a vector for invasive species through adhesion to their surfaces and is transported together with plastics by currents (Gregory 2009). Colonisation processes could occur from the biofouling of plastic items by different taxonomic groups such as molluscs, polychaetes, bryozoans, and hydrozoans, serving not only as a potential vector for the transfer of invasive or alien species but also as a pathway to increase the sedimentation rate of marine plastics to areas of the seafloor (Cózar et al. 2017; Rech et al. 2018; Zettler et al. 2013). In terms of economic costs, marine litter can affect the fisheries and aquaculture industries both directly (i.e., cost of repairs from damage caused by marine litter) and indirectly (i.e., ghost fishing) (Newman et al. 2015). In terms of removal costs, the Balearic Islands (Spain) local government has a sea cleaning boat service that costs an estimated 1 million euros annually to remove floating litter in coastal areas, and in 2019, the service removed ~ 66,000 kg of floating litter (roughly half being plastic), highlighting the success of some management efforts to mitigate marine litter already at sea (ABAQUA 2019).

Therefore, this study is aimed at identifying floating plastic in the marine protected area of Cabrera Archipelago Maritime Terrestrial National Park. The specific aims of the research are (i) to quantify the abundance of microplastic and mesoplastic items along the sea surface through manta net surveys, (ii) to quantify the abundance of macro litter on the sea surface via visual observations, (iii) to assess simultaneously observations of microplastic and macro litter surveys, and (iv) to evaluate the influence of environmental conditions in abundance distribution of micro- and meso-plastics and floating macro-litter.

## Materials and methods

### Study area

**Fig. 1:**
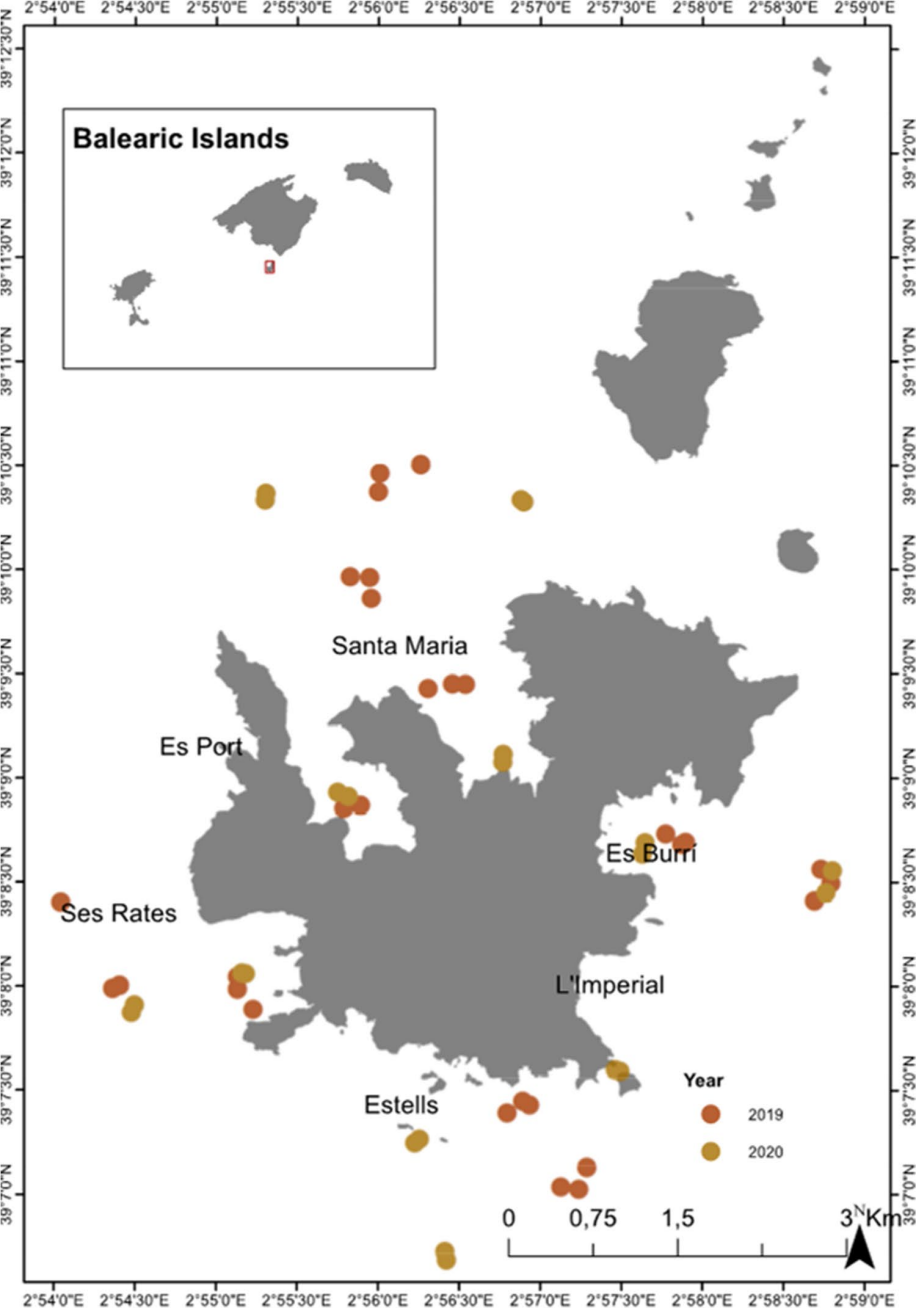
Map of the study area indicating the location of the samples for microplastic and macro litter surveys. Dark orange circles indicate microplastic samples collected during the 2019 survey, and dark yellow circles indicate the simultaneous microplastics and macro marine litter surveys from 2020

Cabrera Archipelago Maritime-Terrestrial National Park (Cabrera MPA hereafter) is located ~ 5km off the southeast coast of Mallorca in the Archipelago of the Balearic Islands (Spain, western Mediterranean Sea) (Fig. 1). Cabrera MPA is a national park and is currently managed by the local government of the Balearic Islands. The main wind regimes are dominated by northeast winds during the summer months and southeastern winds during the winter months (Jansà and Guijarro 2020). In order to study floating litter in this protected area, samples were collected at six sampling sites: Es Port, Santa Maria, Es Burrí, Estells, and Ses Rates (Fig. 1), which have different levels of protection according to the park’s legislation. In Es Port, night and day anchoring is permitted at designated buoys, while in Es Burrí, only daytime anchoring is permitted with previous permission. At Santa Maria, Estells, and Ses Rates sites, anchoring and sailing are prohibited. Two to three transects were performed at each sampling site at two different distances, the first samples were collected parallel to the coast as close as possible (coastal), and the second set of samples was collected offshore at a distance larger than 500 m from the coast (offshore).

### Microplastic assessment

#### Microplastic surveys

Manta net tows were used to conduct 52 microplastic surveys on the ocean’s surface between July and August of 2019 and 2020 onboard the small-scale Research Vessel (RV) Enfú (5.75 m length; 0.46 m draught) from the Centro Oceanográfico de Baleares (IEO, CSIC). Of these, 26 surveys were offshore (at least 500 m from the coast) and 26 were coastal (parallel and as close to the coast as possible). The net used was a standard Hydro-bios manta net with a mesh size of 335 μm. It was equipped with a frame opening of 40 cm in width by 70 cm in length, and the cod length of the net was 260 cm. At each sampling station, the manta net was towed for an average of 15 min at an average speed between 1.5 and 2 knots from the boat’s side and with sufficient distance to ensure that the sampling occurred outside of the turbulence of the wake produced by the vessel’s engine (Fagiano et al. 2022). A Garmin GPS Etrex 10 was used to track the entire track, and the length of each survey was calculated in ArcGIS. Additionally, at each of the stations, the following environmental conditions were recorded in situ: wind intensity, wind direction, and sea state classified according to the Beaufort scale. The net was thoroughly washed from the outside with seawater to collect the organic and inorganic material retained at the cod end. Samples collected were stored in 70% ethanol for further analysis in the laboratory.

#### Laboratory analysis

Once in the laboratory, the collected samples were separated into two aliquots using a Folsom corresponding to 50% of the total sample (in volume). The corresponding aliquots were wet sieved into three size classes. Within the framework of this study, floating litter items were categorised as microplastics (MP) defined here as particles with a diameter (measure of the longest diagonal) smaller than 5 mm, mesoplastics (mesoP) ranging from 5 mm and 2.5 cm, while floating marine macro litter (ML) is defined as litter particles with a diameter larger than 2.5 cm (European Commission 2013; GESAMP 2019; Viršek et al. 2016). Additionally, MP items were classified into two sizes: small microplastics (SMP, 0.33–1.00 mm) and large microplastics (LMP, 1.01–5 mm). The SMP size fraction was filtered through a fibreglass filter (1.2-μm pore size) using a vacuum filtration ramp, while all items in the LMP and mesoP sieves were visually inspected and transferred to clean glass Petri dishes. The samples were then dried at 60 °C and weighed. The items were classified according by their shape (fragments, films, filaments and ropes, pellets, and styrofoam) and colour (white-transparent and opaque, black, blue, red, and other) following Fossi et al. (2019).

For polymeric identification of the items collected, a subset of 10% of the items were randomly selected and analysed by attenuated total reflection Fourier transform infrared spectroscopy (ATR-FTIR, Tensor 27 spectrometer) (Hanke et al. 2013). Filters of the smallest fraction were directly analysed using an ATR crystal attached to a microscope (micro-FTIR). The wavenumber range of 400 to 4000 cm^-1^ was used for measurements, and eight scans per item were performed. Each spectrum was compared using Opus 6.5 software with spectra from a custom polymer library integrating different databases (Löder and Gerdts 2015; BASEMAN D1_2 FTIR reference database, 2021) and an in-house library generated with virgin and weathered reference polymers that include various natural and synthetic materials. Only samples with a hit quality index > 700 (max. 1000) were accepted as confirmed polymers (Fagiano et al. 2022).

To reduce contamination during laboratory analyses, several quality control procedures were carried out. Each researcher wore a white cotton lab coat, and after each use, all sampling equipment was cleaned with filtered distilled water while the workspaces were pre-cleaned with 70% ethanol. Throughout the filtration procedure, a fibre glass filter was present in a glass Petri dish with each SMP sample fraction. During the visual classification of all samples, an open and empty Petri dish was kept nearby. In this study, only fibres were found in the controls, and since fibres were not taken into account in this study, no corrections were made.

### Macro litter surveys

Twenty-two floating marine litter (ML) visual surveys were performed simultaneously with the manta net surveys in 2020. Of these, 11 surveys were offshore and 11 coastal. Observations were made during daylight hours with an observer on the bow of the RV Enfú, and a fixed strip width was set at 3 m perpendicular to the bow and conducted on the glare-free side of the vessel for an average of 15 min at a height of ~ 1 m (Fossi et al. 2019). The Garmin GPS Etrex 10 was used to track the monitored transect and to mark the location of each litter observation.

To calculate the abundance of floating ML items, the total number of observations for each transect was divided by the effective surveyed area using the following equation (Campanale et al. 2019): *D*_*i*_ *= n*_*i*_/(*L*_*i*_ *× W*_*i*_), where *D*_*i*_ is the final abundance calculated, *n*_*i*_ the number of items observed in a transect *i*, *L*_*i*_ is the length of the transect *i*, and *W*_*i*_ (3 m) is the fixed width distance of the strip transect. In terms of the classification of each of the items, all ML observations were classified according to the Joint List of Litter Categories for Marine Macrolitter Monitoring (JL) (Fleet et al. 2021).

### Data analysis

A Kruskal-Wallis analysis was performed to determine differences in MP densities between localities and a Mann-Whitney analysis to determine differences between the plastic abundance of coastal and offshore samples. Pearson’s correlation was used to determine the relationship between the abundances of MP and ML at each sampling location. To determine the environmental effects on the distribution of the items observed during the surveys, a generalised linear model was performed for both net tows (items/km^2^) and visual surveys (items/km^2^) including wind velocity (m/s), sea state (Beaufort scale), and wind direction (cardinal direction). For the net tows model, the response variable was logarithmically transformed to ensure normality, and it was assessed by inspecting the residuals of the model. To identify which predominant winds affect the distribution of microplastics in the coastal region, data for the abundance and weight of items collected with the net tows and the abundance of marine litter from visual surveys were standardised to compare measurements with different units. All net tow values (abundance and weight of items collected per area surveyed) and visual surveys were rescaled from 0 to 1, with 0 being an indication of low abundance and 1 an indication of high abundances. Significance was established at *p* < 0.05. All analyses were performed in R Core Team (2021).

## Results

### Microplastic abundances

**Fig. 2:**
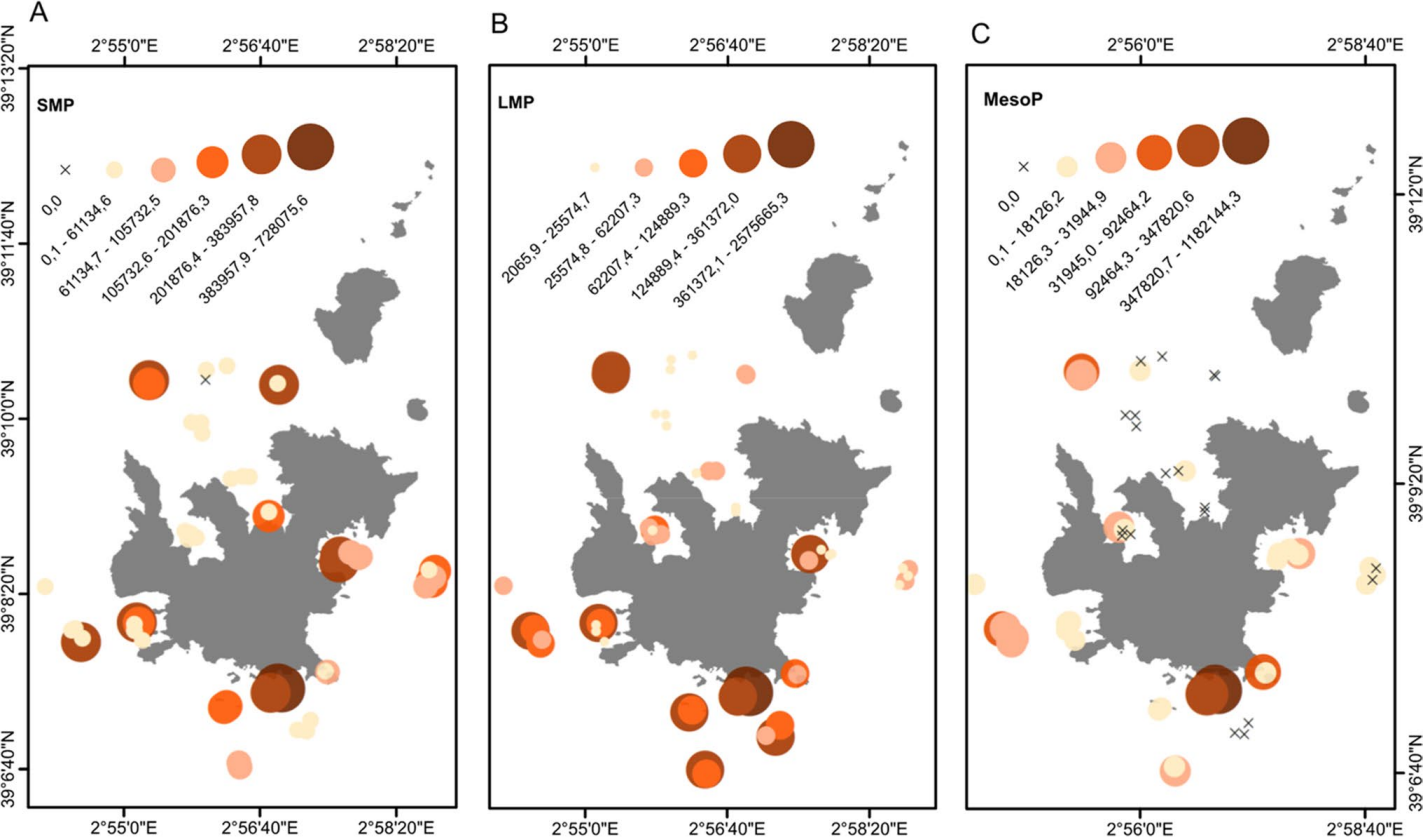
Sample locations for the abundance of marine microplastics quantified in 52 manta net tows for each of three plastic size classes. **A** Small microplastic items/km^2^ (SMP 0.3–1 mm), **B** large microplastic items/km^2^ (LMP 1–5 mm), and **C** mesoplastic items (mesoP 5–25 mm). Data clustering for each category is based on Jenks natural breaks

A total of 52 MP sea surface surveys were carried out, 30 manta net tows in 2019 and 22 manta net tows in 2020, and an average of 328,204.8 ± 785,529.1 items/km^2^ was observed in all samples. From the surveys in 2019, an average abundance of 381,244.4 ± 1,031,082.8 items/km^2^ while in 2020, an average of 255,878 ± 144,676 items/km^2^ were observed and significant differences were found between each year (MW, *p* < 0.001). By location, the sampling location in Estells, in the south, had the highest densities with an average of 1,089,125.41 ± 1,614,878.40 items/km^2^. These highest abundances were concentrated in three samples, and without considering these samples, the overall average abundance decreased to 159,345.7 ± 142,076.6 MPs/km^2^. The location with the lowest abundance was within the bay of Santa Maria where sailing and anchoring are strictly prohibited in the northern region of Cabrera with an average abundance of 71,037.35 ± 72,378.65 MPs/km^2^. The SMP fraction had an average size of 110,984 ± 148,769 items/km^2^, while the LMP fraction had an average size of 158,607 ± 452,528 items/km^2^ (Fig. 2A, B). The mesoP fraction had the fewest items per km^2^, averaging 58,613 ± 206,793 items/km^2^ (Fig. 2C). All sites measured an average weight of 927.1 ± 2731.4 g/km^2^, while the samples from 2019 and 2020 averaged 1,219.7 ± 3,515.6 g/km^2^ and 527.9 ± 872.4 g/km^2^, respectively.

#### Classification

**Fig. 3:**
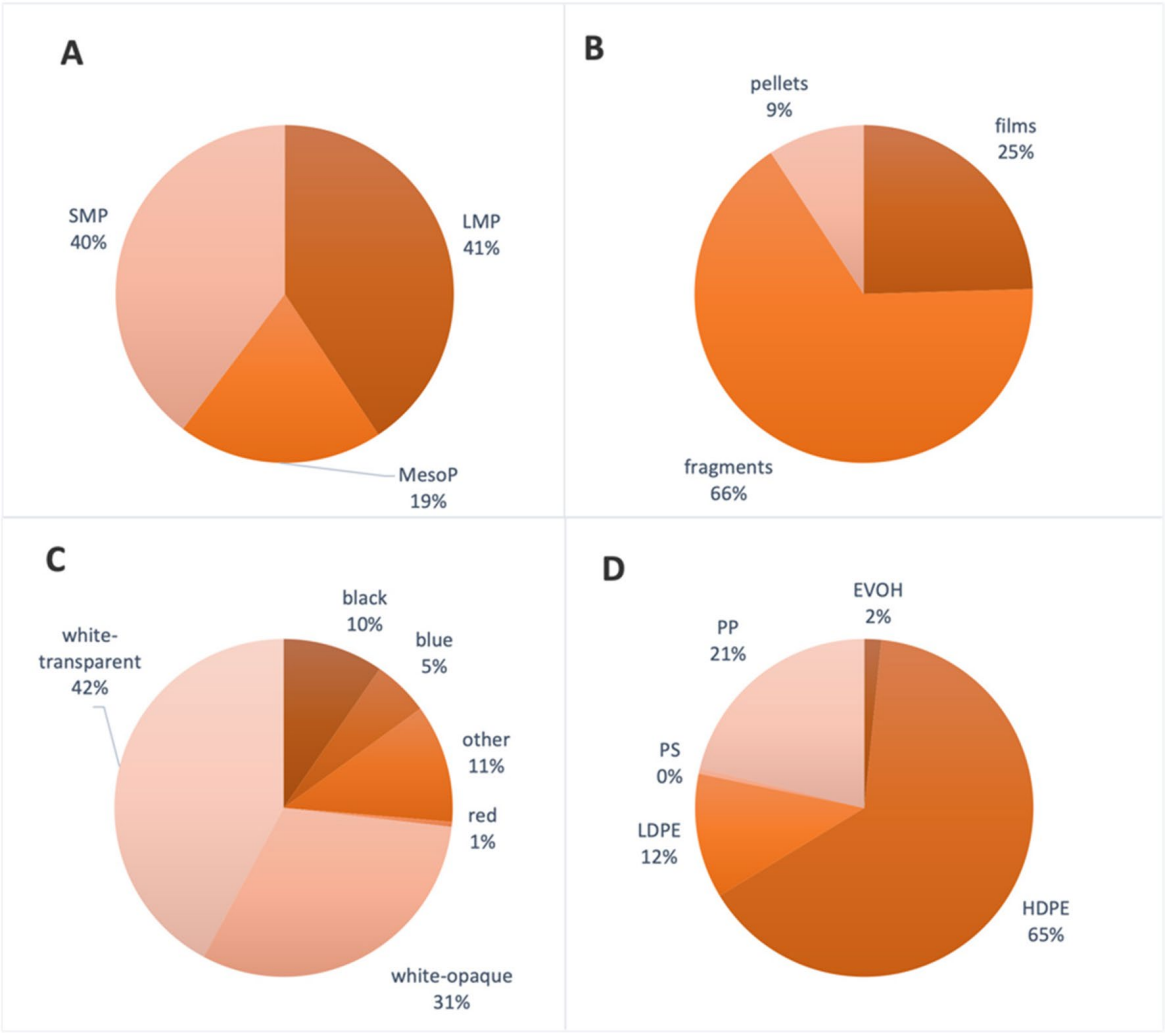
Summary of the characteristics of the microplastic items collected for size (**A**), type (**B**), colour (**C**), and polymer characterisation (**D**)

A subset of 1060 elements was classified into three size classifications, SMP (40%), LMP (41%), and mesoP (19%) (Fig. 3A). In terms of the type of items, the primary type of items collected was fragments (66%), followed by films (25%) and pellets (9%) (Fig. 3B). Although microfibers were not included in this study, they represented 58% of items found in the samples in 2020. For the colours, white-transparent (42%) and white-opaque (31%) were the most common items found (Fig. 3C). In terms of polymer characteristics, a total of 185 items were successfully analysed out of the characterised. Most of the items were high-density polyethylene (HDPE; 65%) followed by polypropylene (PP; 21%) and low-density polyethylene (LDPE; 12%) (Fig. 3D). Other polymers, such as ethylene-vinyl alcohol (EVOH) and polystyrene (PS), accounted for less than 3% of the total items.

### Floating marine macro litter

**Fig. 4:**
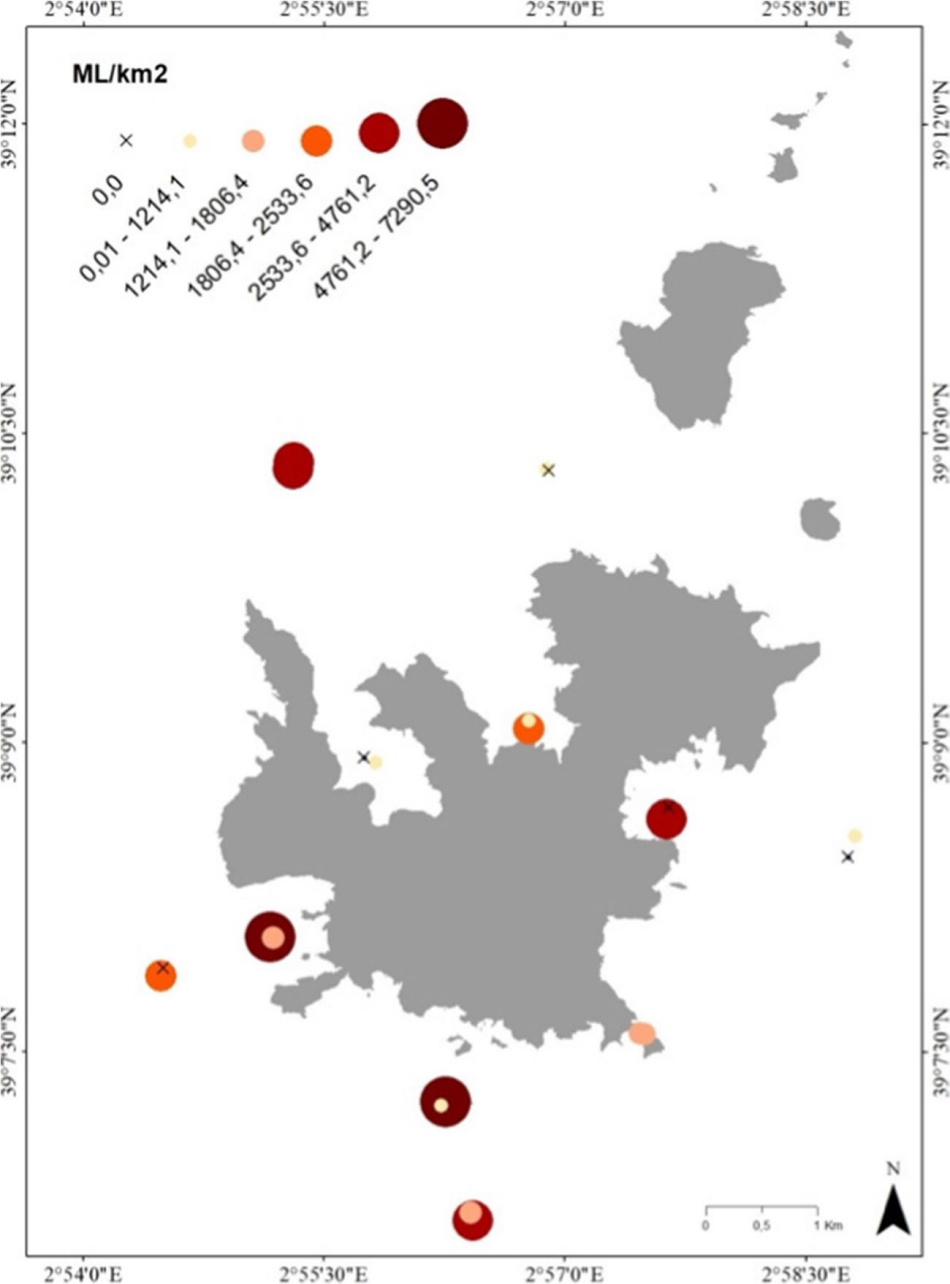
Sample locations for the abundance of floating macro marine litter (ML/km^2^) around Cabrera MPA. Locations were no ML items were observed are represented with an x symbol on the map

Floating ML surveys were performed simultaneously during the 2020 campaign to the microplastic manta trawl surveys. A total of 90 items were observed with an average of 2028 ± 2084 ML items/km^2^ identified from the 22 surveys (Fig. 4). By location, the lowest number of items were found within the bay of Santa Maria on the north shore with an average of 937 ± 905 items/km^2^, and the highest average value was observed in the southern region of Estells with an average of 3473 ± 3137 items/km^2^ observed.

**Fig. 5:**
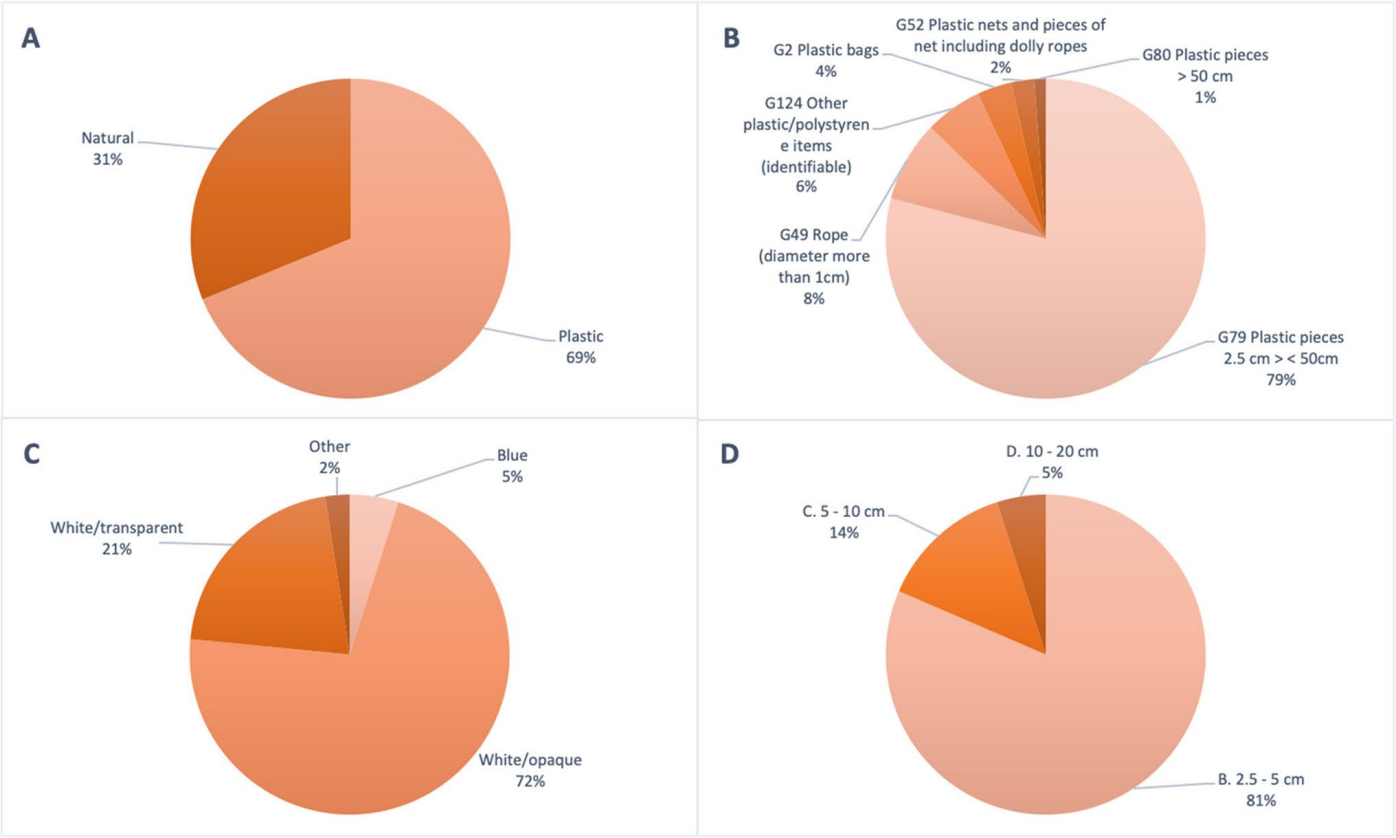
Percent summary of the contribution of each for natural vs. plastic items (**A**), type of items classified according to the Joint List of Litter Categories for Marine Macrolitter Monitoring (JL) (**B**), colour (**C**), and size contribution for all items (**D**) (Fleet et al. 2021)

ML items were observed at 17 of the 22 surveys performed. During visual surveys, items of natural (31%) and plastic (69%) ML were observed, with plastic items being the most abundant (Fig. 5A). The amount of natural litter was similar to a previous study in which 22% of the floating material observed was of natural origin (Suaria and Aliani 2014). The majority of the natural material observed was bamboo, *Posidonia oceanica* leaves, or pieces of *Cymodocea nudosa*. Of the plastic items observed, only six categories from the joint list (JL) were observed. Most of the items were small plastic pieces (G79, 79%) followed by ropes (G49, 8%) and other plastic/polystyrene items (G124, 6%) (Fig. 5B). The remaining three categories were plastic bags (G2, 4%), plastic nets and net pieces (G52, 2%), and plastic pieces > 50 cm (G80, 1%) (Fig. 5B). In addition to the JL classification, items were categorised by colour and size. In terms of colour, the most common were white/opaque (72%) followed by white/transparent (21%) while blue and other combined colours were the least common (7%) (Fig. 5C). Finally, in terms of size, only three size classes of 2.5–5 cm (B, 81%), 5–10 cm (C, 14%), and 10–20 cm (D, 5%) were identified, with the smallest size class (B) found to be the most abundant (Fig. 5D).

### Microplastic and macro marine litter comparisons

**Fig. 6:**
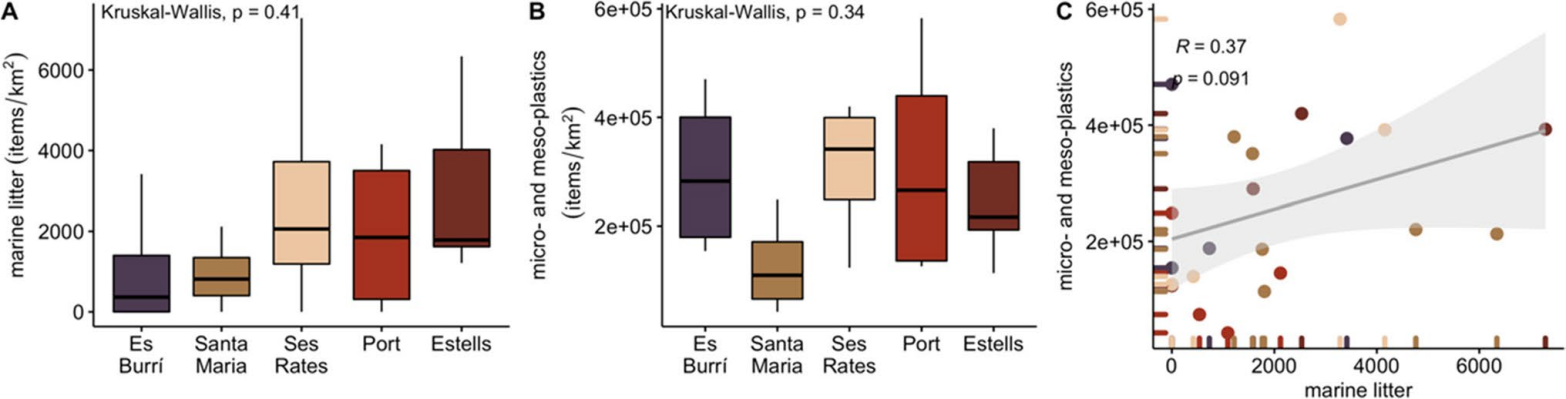
Boxplots of floating macro litter densities by location (**A**) and microplastic abundances by location (**B**) and summary of the results of the simultaneous sampling of microplastic and macro litter for the regression model (**C**). Colour is location dependent (Es Burrí (dark violet), Santa Maria (brown), Ses Rates (light brown), Port (red), and Estells (dark red)

In 2020, 22 MPs and ML surveys were performed simultaneously. The results of Pearson’s correlation did not indicate a significant correlation (*R* = 0.37, *p* > 0.05) between the MP and ML observations (Fig. 6A). No significant differences were observed between the locations for the ML (Fig. 6B; KW, *p* > 0.05) or for the MPs (Fig. 5C; KW, *p* > 0.05). In terms of location, the bay of Santa Maria had the lowest abundances of MPs (127,456 ± 91,603 items/km^2^) and ML (2028 ± 2084 items/km^2^) (Fig. 6C).

**Fig. 7:**
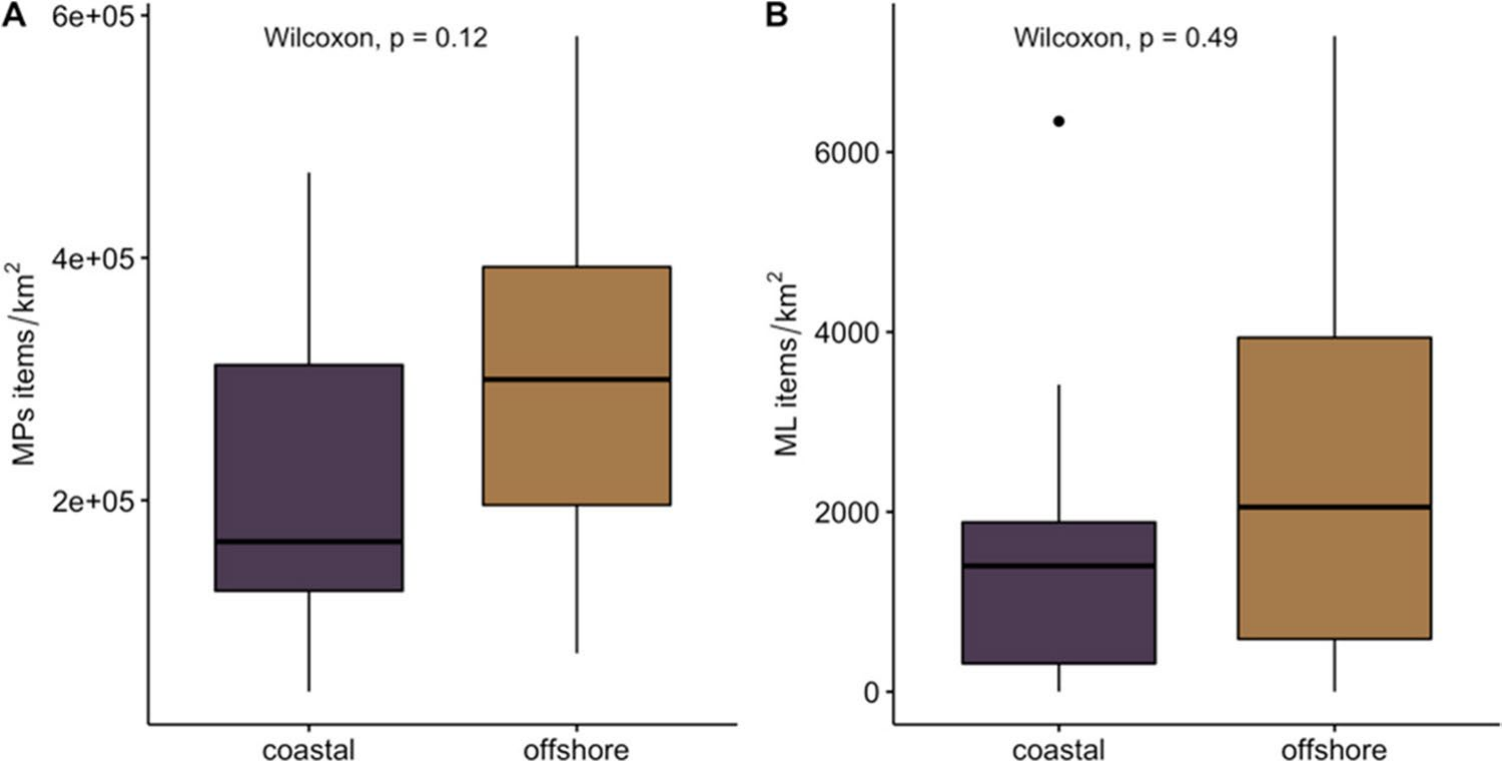
Summary of the abundances of the results from the simultaneous sampling of **A** microplastics (MPs) and **B** macro litter (ML) for the coastal (dark purple) and offshore (light brown) areas of Cabrera MPA

Regarding coastal distances in Cabrera MPA, fewer items were found in coastal samples compared to in the offshore sample for both surveys (manta net for MPs and visual for ML) although they were not found to be significant (MW, *p* < 0.05). For MP items, an average of 217,186 ± 132,146 items/km^2^ was observed in coastal areas, and an average of 302,309 ± 152,002 items/km^2^ was observed in offshore regions (Fig. 7A). A similar pattern was observed for the ML items with an average of 1646 ± 2084 items/km^2^ observed in the coastal waters of Cabrera MPA and an average of 2486 ± 2398 items/km^2^ in the offshore waters of the MPA (Fig. 7B). In terms of the relationship between macro litter items in the coastal and offshore locations, although more items were found in offshore waters, no significant differences were observed between coastal and offshore samples (MW, *p* > 0.05). A similar trend was observed for the MP items where no relationship was found between the abundance quantified in coastal and offshore locations around Cabrera MPA (MW, *p* > 0.05).

### Environmental conditions

**Fig. 8:**
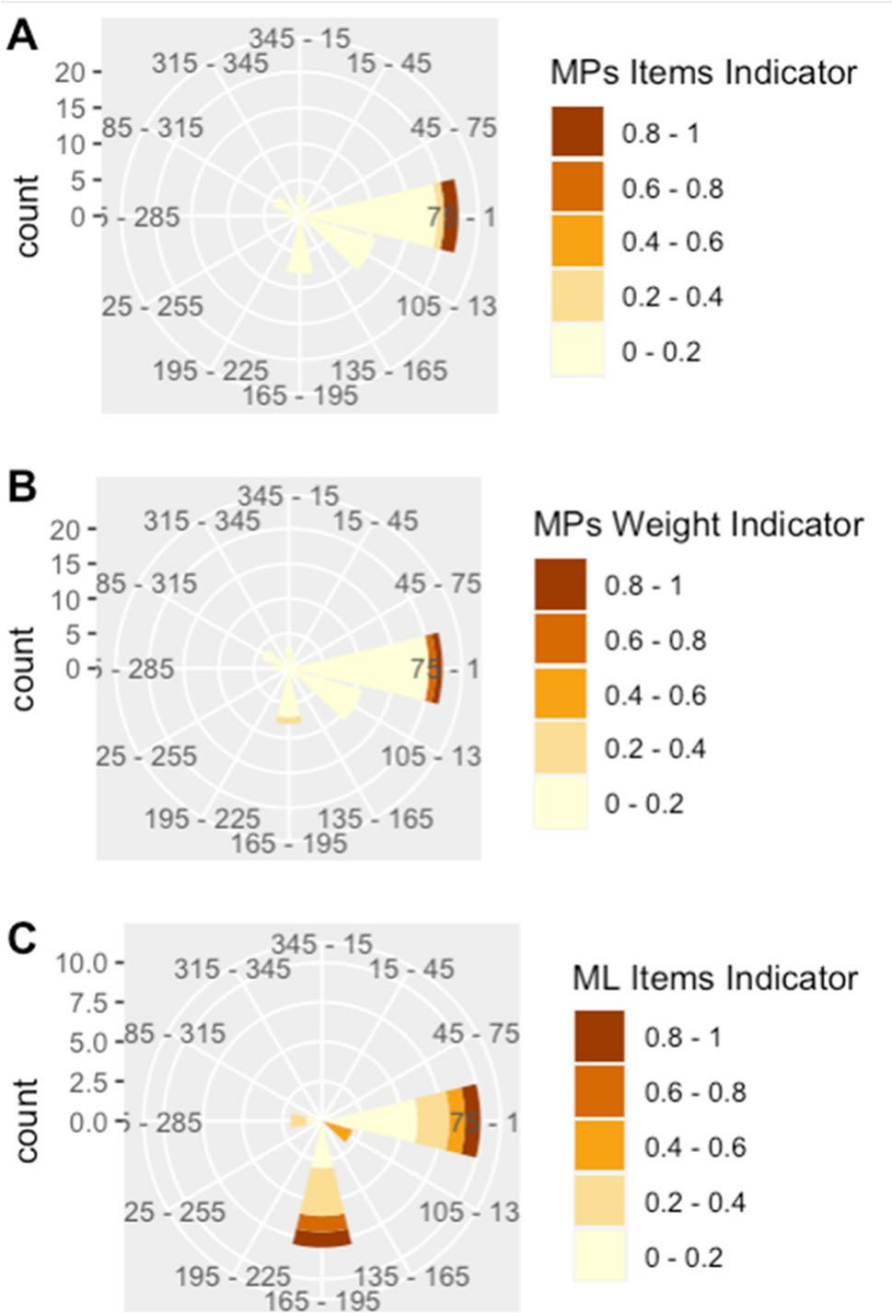
Windrose indicator for microplastic (MP) surveys by number of items (**A**), MP surveys by weight of items (**B**), and floating marine macro litter (ML) surveys by items (**C**). All abundances have been rescaled from 0 to 1 for comparison

For the wind rose indicators generated by rescaling observations for each of the sampling surveys for MPs (items and weight) and ML (items) from 0 to 1, for all sampling activities, eastern wind directions were dominant for both the number and the weight of MP items (Fig. 8A, B). For the MP items, all wind directions had an indicator between 0 and 0.2 except for eastern winds which had indicators 0.2–0.4 and 0.8–1 (Fig. 8A). A similar pattern was observed for the weight of the MPs items; however, in the southern winds, the indicator 0.2–0.4 was also present and for the eastern winds; in addition to the 0–0.2 and 0.8–1 indicators, the 0.6–0.8 indicator was also present (Fig. 8B). For the ML surveys, there was more variability in the eastern and southern winds, while the southeastern winds had an indicator of 0.4–0.6 (Fig. 8C).

**Fig. 9:**
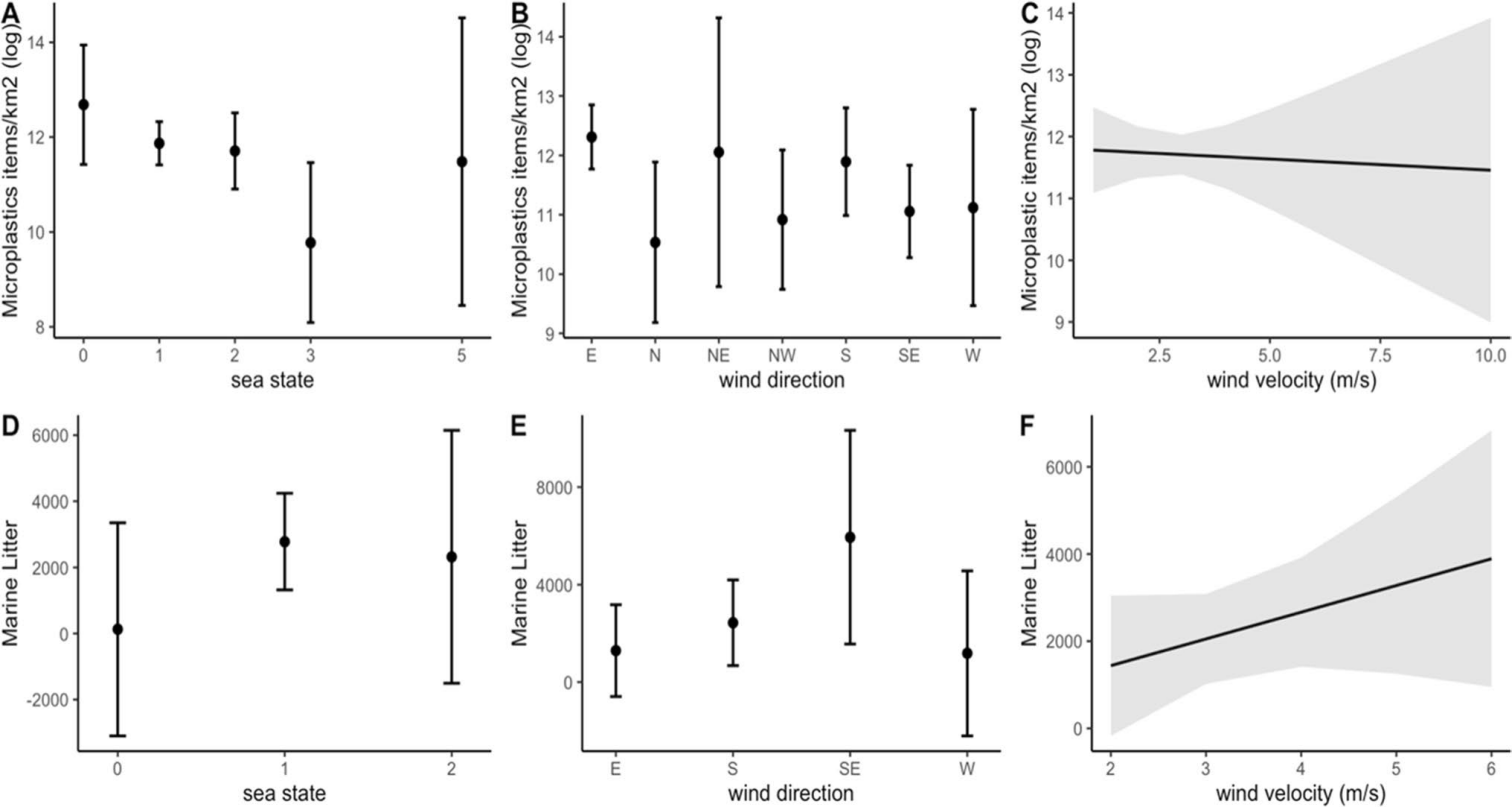
Summary of the marginal effects of the generalised linear models for microplastic surveys (*n* = 52) considering **A** sea state, **B** wind direction, **C** wind velocity and floating marine litter surveys (*n* = 22), **D** sea state, **E** wind direction, and **F** wind velocity

In terms of determining oceanographic conditions that may influence the distribution of MPs and ML on the sea surface, two models were run. The GLM of the MP model (*n* = 52) explained more than 51% of the deviation, and wind direction and sea state were significant predictors (Table 1A, Fig. 9A–C). In terms of sea state, significant differences were found for Beaufort scale 3 and wind direction; significant differences were found between N, NW, SE, and E winds (GLM, *p* < 0.05). Finally, there was no significant effect of wind velocity on MP abundance. In terms of the GLM model for ML, no significant relationships were found between sea state, wind direction, and wind velocity (Table 1, Fig. 9D–F). For the sea state, the highest ML abundances were found during a light breeze with SE winds (Fig. 9D, E). For wind velocity, more ML items were predicted to be observed during higher wind speeds (Fig. 9F).

**Table 1:** Summary of the results of the generalised linear models for microplastic (MP) surveys (A, *n* = 52) and floating marine litter (ML) surveys (B, *n* = 22) considering the sea state according to the Beaufort scale, wind direction (cardinal directions), and wind velocity (m/s)

	Estimate	Std. error	*t* value	Pr (> |*t*|)	
A) MP					
(Intercept)	13.37791	0.81026	16.511	< 2e−16	***
Sea_state 1	− 0.81447	0.67618	− 1.205	0.2355	
Sea_state 2	− 0.97583	0.6876	− 1.419	0.1636	
Sea_state 3	− 2.90766	1.11099	− 2.617	0.0125	*
Sea_state 5	− 1.20084	1.68416	− 0.713	0.48	
N	− 1.77421	0.71601	− 2.478	0.0175	*
NE	− 0.25473	1.13243	− 0.225	0.8232	
NW	− 1.39098	0.64827	− 2.146	0.038	*
S	− 0.41565	0.55314	− 0.751	0.4568	
SE	− 1.25245	0.50594	− 2.475	0.0176	*
W	− 1.1882	0.85186	− 1.395	0.1708	
Wind velocity	− 0.03607	0.16819	− 0.214	0.8313	
B) ML					
Sea_state 1	2650.6	1859.2	1.426	0.1775	
Sea_state 2	2190.3	2129.9	1.028	0.3225	
S	1144.8	1305.6	0.877	0.3965	
SE	4656	2454.3	1.897	0.0803	
W	− 109.3	1652.3	− 0.066	0.9483	
Wind velocity	613.9	466.2	1.317	0.2106

## Discussion

In this present study, the results once more highlight the pervasiveness of plastic from the small microplastic size class to large marine litter items and its high spatial. These findings are in alignment with previous research that examined the ecosystem and the high amount of plastic in the Atlantic Ocean in shoreline strandings, on the sea surface, in the water column, with images of the seafloor, and in the food web, from primary consumers, through mid-trophic fish and seabird top predators (Barnes et al. 2018). In the Canary Islands located in the Atlantic Ocean, microplastic items were observed on all of the 125 beaches surveyed, and in some beaches, these abundances were extremely high (Baztan et al. 2014), while in a MPA in India, although abundances were lower than in non-MPA areas, plastic was continuously the most common observed item (Baroth et al. 2022). Additionally, a more recent model highlights the connectivity between MPAs with larger plastic items travelling longer distances than smaller microplastics (Hatzonikolakis et al. 2022). Overall, the results of this study and previous studies highlight that plastic debris is pervasive and accumulating in marine protected areas around the world.

### Floating microplastics

Floating MPs were found in all of the 52 samples collected around the coastal waters of Cabrera MPA. In terms of the weight of the items, quantities were similar to those observed within the nearby MPA of the Menorcan Channel where an average weight of 1260.6 ± 4089.8 g/km^2^ was identified during autumn months and the number of items ranged between 138,293 items/km^2^ in autumn to 347,793 items/km^2^ during spring (Ruiz-Orejón et al. 2019). In terms of the overall amount of MP items floating along the sea surface, Eriksen et al. (2014) estimated that 92% of all items along the sea surface were microplastics < 5mm. In this study, we observed slightly lower amounts of items < 5mm with an estimated 81% of the items being within the smallest size fraction observed 5–25 mm (Fig. 3A).

In regard to the polymeric composition of microplastics, the majority of the items were of HDPE and LDPE followed by PP. This is in agreement with previous studies where these are the most common particles found floating along the sea surface within the Balearic Islands (Compa et al. 2020) and throughout the Mediterranean Sea (Suaria et al. 2016). All of these polymers are less dense than Mediterranean Sea water, making them some of the most common plastic material on the sea surface (Waldschläger and Schüttrumpf 2019). In the water column, in Rios-Fuster et al. (2022), microplastics quantified within the study area were mainly composed of LDPE and PP (20% each) followed by cellulose acetate (CA) (16%) and PS (14%). In Digka et al. (2018), the authors highlight the similarity in polymeric composition in different compartments where PE and PP were consistently the most common polymers found along the sea surface, in beach sediment and in biota, although in biota, other polymers were also found such as PTFE in mussels and PET, PS, and PTFE in fish. On the seafloor, however, Mistri et al. (2020) reported a variety of polymer types with PE, polyurethane, nylon, and PET being the most predominant from seafloor sediments in the Tyrrhenian Sea. Considering the differences found in polymer types dependent on marine compartments, more studies regarding polymer types are needed to further understand their fate and identify potential sources.

### Floating macro litter

In terms of floating macro litter observations, most visual surveys until now have been done from ships of opportunity such as cruise ships and ferries (e.g., Arcangeli et al. 2017). Observations of floating ML abundances from large vessels, such as those previously mentioned, have provided further information regarding the distribution and abundance of the ML size fraction (Campanale et al. 2019; Suaria and Aliani 2014), and this is especially important when assessing the relation between the overlap of marine species such as cetaceans with litter (Di-Méglio and Campana 2017). Considering this, Vlachogianni et al. (2017) highlights a major constraint in sampling macro litter densities from ships, which might affect the accurate estimation of the observation width and the loss of detection ability for the smaller sizes with increased observation height and vessel speed. Palatinus et al. (2019) highlights advantages of using smaller boats, considering the reduced distance from the observer to the sea surface, giving the observers more opportunities to detect smaller items. This was evident in this study as the majority of the items (81%) belonged to the smallest size fraction observed (Fig. 5D, 2.5–5 cm), and in a similar study in the Black Sea, 96% of floating marine macro litter observed was mostly plastic and within a similar size range of 2.5–10 cm (González-Fernández et al. 2022). In agreement with previous studies (Palatinus et al. 2019; Vlachogianni et al. 2017), it was expected that the ability to detect small-sized items (2.5–5 cm) would increase when surveying with small vessels at low velocities instead of surveying with larger vessels and at higher heights from the sea surface. Moreover, for the ML surveys, Campanale et al. (2019) observed a range from 0 to 9205 items/km^2^ and with a mean density of 492 items/km^2^ across the study areas in the western Mediterranean Sea, which is within the range reported in this study (0 to 7290.5 items/km^2^). Results from this study further confirm the importance of establishing a realistic size limit of observations, and considering the alarmingly high densities of ML within the coastal area of Cabrera MPA, we would recommend future studies to use small vessels for monitoring marine litter when possible to adequately quantify the densities of ML floating along the sea surface, especially with the manta net as we quantify higher abundances of litter.

No discernible differences were found between the offshore and coastal samples in this study, despite the fact that the offshore samples had considerably higher densities. Although the diversity of polymers was greater in the concentrations of plastic within the first kilometre from the coastline, previous studies such as Vlachogianni et al. (2018) have shown that there was an increasing gradient in the concentrations of marine litter with increasing distances from the coast. In a more recent study, one of the main drivers for higher abundances in coastal areas was a high fractal dimension of the coastline attributed to a more complex coastal zone (Compa et al. 2020). This was also observed in Brennan et al. (2018) where the coastal shape influenced the accumulation of marine litter on the shoreline, and these areas consisted of a high arrival rate and a low departure rate, inducing accumulation. Considering our observations and that although for this study they were considered offshore, both sampling areas were still within 2 km of the coastal area, and future studies should consider visual observations further out at sea. In this sense, guidelines can be taken from the recent technical report from the Joint Research Council which compiles the existing information regarding techniques and options for monitoring floating ML at sea, providing a comprehensive analysis of the different methods used to date and the optimization dependent on survey locations and resources available (Vighi et al. 2022).

### Simultaneous surveys

In this study, no significant differences were found in the ML and MP surveys between locations neither between coastal and offshore areas of Cabrera MPA. This is important to highlight, as this indicates that despite potential variability in densities, both ML and MP are found regardless of their protection status but also regardless of the predominant wind and the influence of oceanographic factors such as currents and waves. A numerical study within the harbour of Cabrera MPA highlighted the residence time of particles to be about 8.7 days and had a determinant factor over the biological communities in this area (Orfila et al. 2005). At a more regional scale, Cabrera MPA is situated north of the Algerian Current and the Algerian Gyre, which might have an influence in the transport of items for other regions in the Mediterranean considering that some minor intrusions of new Atlantic waters were observed between the islands of Cabrera and Menorca (Balbin et al. 2014).

In terms of ML and MP surveys, the simultaneous surveying indicated that less than 1% of the items was observed during the ML visual surveys. Here, we highlight two important observations. The first being the presence of items in coastal areas could potentially be more abundant than ML and MPs at sea, and the second being that MPs surveys collect a high percentage of items that are within the mesoP range, which may not observed during the ML strip surveys due to their small size. In a recent study, numerical models have identified that the coastal zone may potentially act as a trapping area for ML (Onink et al. 2021). Therefore, it is complimentary and recommended to perform both surveys simultaneously to achieve a complete monitoring of the floating marine litter.

Few studies so far have performed simultaneous MP and ML sea surface surveys. In our study area, a positive correlation was found between microplastic and macro litter sampling although this was not found to be significant. This is similar to previous research where a low correlation was observed between the abundance of macro and microplastics during the same cruises in the Mediterranean Sea (Suaria et al. 2018). Additionally, with both surveys, all size classes are covered as the MP surveys with surface tows cover size classes up to 25 mm, and ML visual surveys can be used to identify items larger than 25 mm. However, future assessments would benefit from combining simultaneous surveys of microplastic and macro litter, not only for the sea surface but also for the seafloor and other marine compartments (Palatinus et al. 2019).

### Environmental conditions

The majority of the items were probably transported by currents in the case of Cabrera MPA, where human activities are heavily monitored, taking into account the small area monitored in conjunction with the local currents and the fact that the distance to the closest large cities was on the adjacent island of Mallorca. Plastic items floating along the sea surface have long been identified as being transported from other regions. Backtracking simulations by Compa et al. (2020) highlight connectivity in the near-shore areas of the Balearic Islands with other regions in the western Mediterranean Sea. In addition, Liubartseva et al. (2019) highlighted that plastic that ends up on the beaches of the nearby MPA of ses Salines d’Evissa i Formentera (Eivissa, western Mediterranean Sea) comes primarily from the scraps of ships followed by human activities from several major cities on the mainland of Spain (e.g., Alicante, Valencia, Barcelona). Furthermore, Jansà and Guijarro (2020) highlight that Cabrera MPA does not have its own well-developed wind regime, but rather the dominant winds in Cabrera MPA are basically the general winds from the open sea and further highlight the transferability from the open sea to the park. The sea surface circulation resulting in the dispersal of plastics in this region is characterised by the mesoscale phenomena, such as an anticyclonic eddies and the recirculation of the Balearic Current with the convergence of the Northern Current and the entrance of the Recent Atlantic waters (Liubartseva et al. 2019).

Although wind velocity was not a significant factor in the distribution of MP, a negative relationship was also found in previous MP net tows in the region (Compa et al. 2020) where wind-induced mixing from high wind conditions has been attributed to be forcing a vertical distribution of MPs in the water column, resulting in fewer MPs items on the surface layer (Kukulka et al. 2012). Regarding the distribution of prevailing winds, for ML, the highest abundances of items were most common during the southern and eastern winds, the southeastern winds are the most predominant winds in Cabrera MPA and may account for the high marine debris abundances found at the sites in the southern region. In terms of wind intervals of direction and speed (average wind speed and direction in periods of ten minutes) and average wind speed by directions, during the summer months (June, July, and August) of Cabrera, there is a continuous wind fluctuation in all directions; however, the NE-E winds have the highest median interval percentage of winds coming from this direction ranging from 20.1 to 24.2% (Jansà and Guijarro 2020). In this study, the eastern winds account for 75% of the MPs arriving to the coast, further determining the arrival of MPs from the sea during eastern wind events.

It is also important to highlight that the sampling occurred prior to the COVID-19 pandemic and during the following months after that the lockdown measures were beginning to be less restrictive. A significant difference was found between years, with 1/3 fewer items collected during the pandemic season in 2020. During the pandemic, the number of visitors to Cabrera MPA was dramatically reduced due to local mobility and park restrictions, such as no overnight stays in the Cabrera MPA’s hostel. Additionally, mobility restrictions were applied for non-residents during 2020 throughout the Balearic Islands (Mallorca, Menorca, and the Pitiüses) meaning that tourism was reduced to minimum numbers compared to the previous year. In 2019 between organised excursions and recreational boaters and divers, about 82,000 people visited Cabrera MPA (Cortés López et al. 2019). Considering this, it is important to highlight the potential pressure that tourism could have on the marine environment in addition to being a potential source of marine litter.

## Conclusions

Cabrera MPA represents a large diversity in both flora and fauna in terrestrial and marine ecosystems, and the results from this study highlight Cabrera MPA which is exposed to high levels of marine litter in the surrounding coastal waters on the sea surface. In terms of the MPs items, fragments and HDPE were the dominant microplastic type found on the sea surface while the floating ML was one order of magnitude higher than previous studies, indicating the prevalence of these items within the MPA. Smaller vessels were linked to more frequent visual observations of plastic waste in this study, which is another notable finding. The study’s findings highlight the intensity of MPs and ML in coastal waters, increase awareness of the transferability of ML across regions, and provide a baseline for management efforts to reduce floating material in MPAs. This study found that an MPA contained high levels of marine litter, indicating that these areas were more likely to come into contact with lost and discarded plastic marine litter, especially during the summer when there is an increase in human activity in coastal areas.

## Data Availability

Data are available upon reasonable on request.
